# Effects of plyometric jump training on soccer player’s balance: a systematic review and meta-analysis of randomized-controlled trials

**DOI:** 10.5114/biolsport.2022.107484

**Published:** 2021-10-06

**Authors:** Filipe Manuel Clemente, Rodrigo Ramirez-Campillo, Daniel Castillo, Javier Raya-González, Markel Rico-González, Rafael Oliveira, Thomas Rosemann, Beat Knechtle

**Affiliations:** 1 Escola Superior Desporto e Lazer, Instituto Politécnico de Viana do Castelo, Rua Escola Industrial e Comercial de Nun’Álvares, 4900-347 Viana do Castelo, Portugal; 2 Instituto de Telecomunicações, Delegação da Covilhã, Lisboa 1049-001, Portugal; 3 Department of Physical Activity Sciences. Universidad de Los Lagos. Santiago, Chile; 4 Centro de Investigación en Fisiología del Ejercicio. Facultad de Ciencias. Universidad Mayor. Santiago, Chile; 5 Faculty of Health Sciences, Universidad Isabel I, Burgos, Spain; 6 Department of Physical Education and Sport, University of the Basque Country, UPV-EHU, Lasarte 71, 01007 Vitoria-Gasteiz, Spain; 7 Sports Science School of Rio Maior–Polytechnic Institute of Santarém, 2140-413 Rio Maior, Portugal; 8 Life Quality Research Centre, 2140-413 Rio Maior, Portugal; 9 Research Centre in Sport Sciences, Health Sciences and Human Development, 5001-801 Vila Real, Portugal; 10 Institute of Primary Care, University of Zurich, 8091 Zurich, Switzerland; 11 Medbase St. Gallen Am Vadianplatz, 9001 St. Gallen, Switzerland

**Keywords:** Football, Human physical conditioning, Reactive strength, Power, Motor skills

## Abstract

Plyometric jump training (PJT) can be used for improving balance through bilateral and unilateral jump-landing drills. Since the increased number of articles testing the effects of PJT on dynamic and static balance, it is relevant to summarize the evidence and determine the effects across different original articles. This systematic review with meta-analysis was conducted to assess the effects of PJT programs on dynamic and static balance in soccer players. The data sources utilized were Cochrane, Medline (PubMed), SPORTDiscus, and Web of Science. (i) Soccer players of any age or sex without injury, illness, or other clinical conditions; (ii) PJT-based programs restricted to a minimum of three weeks (duration); (iii) passive or active control groups; (iv) pre-post interventions values of dynamic and/or static balance; (v) randomized-controlled trials; and (vi) peerreviewed original full-text studies written in English, Portuguese, and/or Spanish. The database search initially identified 803 titles. From those, eight articles were eligible for the systematic review and meta-analysis. The results showed no significant differences between PJT and active controls in dynamic anterior, postero-medial, or postero-lateral balance for both left and right legs (p > 0.05). Additionally, no significant differences were found between PJT and active controls in terms of static balance (p = 0.495). The current evidence suggests that PJT has no significant advantage over active control groups in terms of dynamic or static balance.

## INTRODUCTION

Soccer is characterized by an intermittent exercise profile in which periods of low- to moderate-intensity activities are interspaced by high-intensity efforts [1]. Among high-intensity efforts, accelerations/decelerations, turns, and jumps are key determinants of duels [2]. Additionally, as soccer is a tactical-technical-based game, actions such as kicking, passing, dribbling, and cutting maneuvers are often performed during matches [3, 4]. To support high performance during the aforementioned actions, players need adequate levels of strength, power, and aerobic capacity [5, 6]. However, excellent balance can also bolster soccer players’ performance. For example, better balance was meaningfully correlated with kicking accuracy [7]. Additionally, jumping performance was associated with better postural control [8], even when balance conditions were poor [9]. Finally, adequate levels of balance might protect against musculoskeletal and ligament injury risk [10]. In one study, greater asymmetries observed in a dynamic balance test were associated with lower extremity injury risk [11].

There are two main dimensions of balance (also named postural control) [12, 13]. Static balance involves the ability to maintain a base of support with minimal movement, and dynamic balance is the ability to perform a task while maintaining a stable position. Balance can be influenced by several factors, including sensory information (e.g., somatosensory, visual, and vestibular systems), joint range of motion, and the neuromuscular control of the athlete [14–16]. Assessments of static balance are often performed by monitoring the center of pressure motion for a specified duration in which the athlete must keep a unilateral or bilateral position on a force platform with eyes open or close [17, 18]. For assessing dynamic balance, some field-based tests are used, namely the Star Excursion Balance test [19] or Y-balance test [20], in which the athlete, while assuming a unilateral stance, tries to reach the farthest point possible with the contralateral limb. Dynamic balance can also be measured in laboratory settings using a stabilometer [21], force platforms [17], or a Biodex balance system [22].

Balance training can improve balance ability [23]. However, other training methods, such as plyometric jump training (PJT), also may improve balance at the same time as jumping (i.e., power), maximal strength, sprinting, and acceleration [24][25–27], thereby providing a time-efficient training method. A typical PJT program incorporates jump drills that leverage the stretch-shortening cycle of the musculotendinous unit [28], commonly through unilateral/bilateral or vertical/horizontal jumps on different surfaces (e.g., stable or unstable) [29]. Such drills may stimulate factors associated with balance ability, such as sensory information, joint range of motion, and neuromuscular control [14–16]. In this sense, PJT might offer adequate stimuli for improving soccer players’ balance [30].

Some (but not all) studies revealed balance improvements in soccer players after PJT. Moreover, most PJT studies conducted among soccer players have incorporated a small sample size (e.g., 10 participants per group). In this scenario, a systematic review of the literature following a meta-analytical approach might contribute to solving controversies regarding the effects of PJT on soccer players’ balance. Therefore, the purpose of this study was to conduct a systematic review with meta-analysis to assess the effects of PJT programs on dynamic and static balance in soccer players.

## MATERIALS AND METHODS

This systematic review and meta-analysis followed the Cochrane Collaboration [31], PRISMA (Preferred Reporting Items for Systematic Reviews and Meta-analyses) guidelines [32] and guidelines for systematic reviews in sports science [33]. The PICOS approach (Population, Intervention, Comparator, Outcomes, Study design) was followed: (P) soccer players from any age or sex, without injury, illness or other clinical condition; (I) PJT-based programmes restricted to a minimum of 3 weeks (duration) and no restricted to frequency (number of sessions per week); (C) Passive or active control groups; (O) Pre-post values of dynamic and/or static balance; and (S) randomized controlled-trials. The protocol was registered with the International Platform of Registered Systematic Review and Meta-Analysis Protocols with the number 202110107 and the DOI number 10.37766/inplasy2021.1.0107.

### Eligibility criteria

Inclusion and exclusion criteria for this systematic review and meta-analysis can be found in table 1.

**TABLE 1:** Inclusion and exclusion criteria

Item	Inclusion Criteria	Exclusion Criteria
Population	Soccer players from any age or sex, without injury, illness or other clinical condition. Futsal (indoor soccer), if any, will be included.	Soccer players in rehabilitation or in return-to-play programmes. Other sports than soccer/futsal (e.g., volleyball, basketball, rugby, Australian or American football).
Intervention	PJT-based programmes (bilateral and/or unilateral) restricted to a minimum of 3 weeks (duration) and no restricted to frequency (number of sessions per week).	Other training methods. PJT combined with other training methods.
Comparator	Passive or active control groups.	Other PJT training group (i.e., PJT vs. PJT without control group were excluded). Cases as two PJT and a control were included.
Outcome	Pre-post intervention values of static and/or dynamic balance.	Outcomes not related to dynamic and/or static balance; no information (e.g., mean; standard deviation) reported for pre- and/or post-intervention (e.g., follow-up excluded)
Study Design	Randomized controlled and/or parallel trials.	Non-randomized and non-controlled studies
Additional criteria	Peer reviewed, original, full-text studies written in English, Portuguese and/or Spanish.	Written in other language than English, Portuguese and/or Spanish. Reviews, letters to editors, trial registrations, proposals for protocols, editorials, book chapters, conference abstracts.

Duplicates were identified using the reference manager software (EndNote^TM^ X9, Clarivate Analytics, Philadelphia, PA, USA). Two authors (MRG and JRG) independently performed screening of the title, abstract and reference list of each study to locate potentially relevant studies. Additionally, they reviewed the full version of the papers in detail to identify articles that met the selection criteria and those that were excluded. A discussion was made in the cases of discrepancies regarding the selection process with the participation of a third author (DC).

### Information sources

Electronic databases (Cochrane, Medline (PubMed), SPORTDiscus, and Web of Science) were searched for relevant publications, from inception up to January 26, 2021. Keywords and synonyms were entered in various combinations in all fields: (“Soccer” OR “Football”) AND (“plyometric*” OR “ballistic” OR “stretch-shortening cyle” OR “reactive strength” OR “jump*”) AND (“balance” OR “stability”). An external expert was contacted to verify the final list of references included in this systematic review and to indicate if there was any study that was not detected through our search.

### Extraction of data

A data extraction sheet, adapted from the Cochrane Consumers and Communication Review Group’s data extraction template [34], was used to assess inclusion requirements and subsequently tested on ten randomly selected studies (i.e., pilot testing). This process was conducted by two independent reviewers (MRG and JRG). Any disagreement regarding study eligibility was resolved in a discussion between both reviewers and a third author (FMC). Full text articles excluded, with reasons, were recorded. The records were registered in a form created in Microsoft Excel (Microsoft Corporation, Readmon, WA, USA).

### Data items

Aiming to establish consistency in data analyzing and reporting, only measures that were analyzed three or more times for different articles were included [25]. The dynamic and/or static balance was chosen as the main outcome. Adverse effects were also extracted as secondary outcome, in case of any reported. The pre- and post-data (e.g., mean, standard deviation, number of participants, per each group) was extracted. Intermediate assessments (in middle of interventions) and/or follow-up periods (without intervention) were not extracted. The method used for the assessment of balance was also extracted. Additionally, the following information was extracted from the included studies: (i) participants age (years), sex, competitive level; (ii) PJT training (only or combined with other method); type of jumps included (unilateral and/or bilateral); (iii) PJT characteristics as: duration (number of weeks); training frequency (sessions per week); intensity level (e.g., maximal); jump box height (cm); number of total jumps completed during the intervention; rest type (active, passive) between sets (min); rest time between repetitions (s); rest time between sessions (hours); type of jump surface (e.g., unstable; stable); training period of the year (e.g., pre-season, in-season).

### Assessment of methodological quality

The Physiotherapy Evidence Database (PEDro) scale was used to assess the methodological quality of the randomized-controlled trials included in this systematic review and meta-analysis. The scale scores the internal study validity in a range of 0 (low methodological quality) to 10 (high methodological quality). Eleven items are measured in the scale. The criterion 1 is not included in the final score. Points for items 2 to 11 were only attributed when a criterion was clearly satisfied. Two of the authors (MRG and FMC) independently scored the articles. Disagreements in the rating between both authors was resolved through discussion with a third author (DC). Aiming to control the risk of bias between authors, the Kappa correlation test was used to analyze the agreement level for the included studies. A minimum agreement level of k = 0.90 was established.

### Summary measures, synthesis of results, and publication bias

We followed previously stablished methods [25, 26]. Briefly, analysis and interpretation of results were only conducted in the case of at least three studies provided baseline and follow-up data for the same measure. Pre-training and post-training means and standard deviations (SD) for dependent variables were used to calculate effect sizes (ES; Hedge’s *g*) for each outcome measure in the PJT and control groups. Data were standardized using post-intervention SD values. The random-effects model was used to account for differences between studies that might impact the PJT-based effect [35, 36]. The ES values are presented with 95% confidence intervals (CI). Calculated ES were interpreted using the following scale: < 0.2, trivial; 0.2–0.6, small; > 0.6–1.2, moderate; > 1.2–2.0, large; > 2.0–4.0, very large; > 4.0, extremely large [37]. Heterogeneity was assessed using the *I* ^2^ statistic, with values of < 25%, 25–75%, and > 75% considered to represent low, moderate, and high levels of heterogeneity, respectively [38]. The risk of bias was explored using the extended Egger’s test [39]. When bias was present, the trim and fill method was applied [40], in which case L0 was assumed as the default estimator for missing studies [41]. All analyses were carried out using the Comprehensive Meta-Analysis software (version 2; Biostat, Englewood, NJ, USA). Statistical significance was set at *p* ≤ 0.05.

## RESULTS

### Study identification and selection

**FIG. 1:**
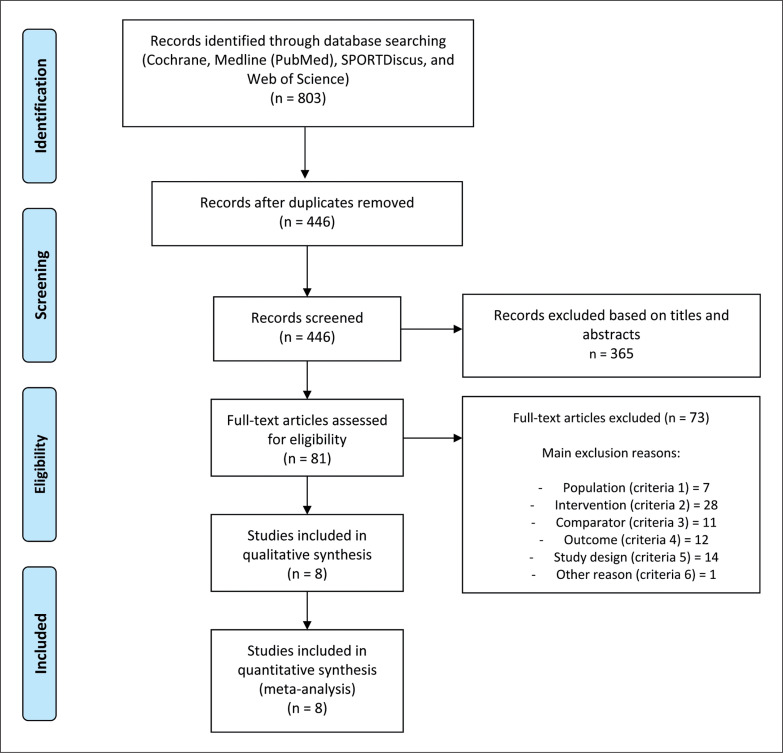
PRISMA flow diagram highlighting the selection process for the studies included in the current systematic review.

The searching of databases identified an initial 803 titles. These studies were then exported to reference manager software (EndNote^TM^ X9, Clarivate Analytics, Philadelphia, PA, USA). Duplicates (357 references) were subsequently removed either automatically or manually. The remaining 446 articles were screened for their relevance based on titles and abstracts, resulting in the removal of a further 365 studies. The full texts of the remaining 81 articles were examined diligently. After reading full texts, a further 73 studies were excluded owing to the following criteria: population (n = 7); intervention (n = 28); outcome (n = 12); comparator (n = 11); and study design (n = 14). Therefore, 8 articles were eligible for the systematic review and meta-analysis (Figure 1). The 8 studies included provided mean and standard deviation post-detraining data for at least the main outcome.

### Study characteristics

The characteristics of the eight studies included in the meta-analysis can be found in Table 2. Additionally, the details of the PJT-based programs can be found in Table 3. The included randomized-controlled studies involved 14 individual experimental groups and 191 participants, and 105 participants in the 8 control groups.

**TABLE 2:** Characteristics of the included studies and outcomes extracted.

Study	N	Mean age (yo)	Sex	Type of control group	Out-comes	Tests used in the original studies	Measure extracted from the tests in the original studies	Tendency
Drouzas et al. [58]	Exp^a^: 23 Exp^b^: 23 ActCon: 22	8–13	M	ActCon: specific program with soccer skills	SB	Flamingo balance test	SB: number of times the participant lost their balance (composite).	SB: less, better.
Hammami et al. [50]	Exp^d^: 12 Exp^e^: 14 PassCon: 12	16.2 ± 0.2 16.3 ± 0.4 16.4 ± 0.2	M	ActCon: continued with the standard training	DB SB	Y-Balance test (anterior, posterior-medial, posterior-lateral directions, plus composite score) Static Stork Balance Test	DB: anterior, posterior-medial, posterior-lateral directions displacement (cm) SB: Stork, time (sec) for right leg	DB: higher, better SB: higher, better
Jlid et al. [47]	Exp: 14 AtcCon: 14	11.8 ± 0.4 11.6 ± 0.5	M	ActCon: continued with their standard soccer training	DB	Star excursion balance test (antero-lateral, anterior, antero-medial, medial, posterior-medial, posterior, posterior-lateral, and lateral)	DB: anterior, posterior-medial, posterior-lateral directions displacement (cm)	DB: higher, better
Jlid et al. [59]	Exp: 14 ActCon: 13	19.0 ± 0.9 19.0 ± 0.7	M	ActCon: continued training without change	DB	Star excursion balance test (antero-lateral, anterior, antero-medial, medial, posteromedial, posterior, postero-lateral, and lateral)	DB: anterior, posterior-medial, posterior-lateral directions displacement (cm)	DB: higher, better
Porrati-Paladino et al. [49]	Exp: 9 ActCon: 8	18–30	F	ActCon: received an intervention using eccentric hamstring exercises	DB	Y-Balance test (anterior, posterior-medial, posterior-lateral directions)	DB: anterior, posterior-medial, posterior-lateral directions displacement (cm)	DB: higher, better
Ramirez-Campillo et al. [57]	Exp^a^: 12 Exp^b^: 16 Exp^c^: 12 PassCon: 14	Exp^a^: 11.2 ± 2.4 Exp^b^: 11.0 ± 2.0 Exp^c^: 11.6 ± 1.7 PassCon: 11.6 ± 2.7	M	PassCon	SB	Normal stance eyes open and eyes closed Perturbed stance, eyes open and eyes closed in a balance platform (Data obtained for anterior-posterior and medial-lateral)	SB: bilateral normal stance eyes open displacement, (anterior-posterior, cm)	SB: less, better
Ramirez- Campillo et al. [24]	Exp^c,h^: 10 Exp^c,i^: 10 Exp^c,j^: 10 PassCon: 10	Exp^c,h^:11.4 ± 2.4 Exp^c,i^:11.6 ± 1.4 Exp^c,j^:11.4 ± 1.9 PassCon: 11.2 ± 2.3	M	PassCon	SB	Normal stance eyes open and eyes closed	SB: bilateral normal stance eyes open displacement, (anterior-posterior and medial-lateral, cm)	SB: less, better
Trecoci et al. [43]	Exp: 12 ActCon: 12	11.3 ± 0.7	M	ActCon: specific program with soccer skills	DB	Lower quarter Y balance test (anterior, posterior-medial, posterior-lateral directions, plus a composite score)	DB: composite score (cm)	DB: higher, better

a : bilateral plyometric;

b : unilateral plyometric training;

c : combined bilateral and unilateral plyometric training group;

d : unloaded;

e : with load;

f : combined agility and plyometric;

g : combined balance and plyometric;

h : vertical plyometric;

i :horizontal plyometric;

j : combined vertical and horizontal plyometric;

l: combined strength, balance, agility and plyometric; M: male; F: female; Exp: experimental group; ActCon: active control group; PassCon: passive control group without experimental approach; SB: static balance; DB: dynamic balance; PHD: prone hold test; ND: not described; plyo: plyometric.

**TABLE 3:** Characteristics of the included studies and outcomes extracted.

Study	BM	H	SPT	Fitness level*	Laterality	Freq	Wk	BH	TJ	Comb	PO	TP
Drouzas et al. [58]	Exp^a^: 39.3 ± 8.2 Exp^b^: 36.1 ± 7.8 ActCon: 38.5 ± 10.6	Exp^a^: 142.2 ± 8.7 Exp^b^: 139.2 ± 7.0 ActCon: 141.6 ± 10.4	No experience	Moderate	Exp^a^: U Exp^b^: B	2	10	10–30 cm	60–120	No	Sets and reps	Middle season
Hammami et al. [50]	Exp^d^: 59.8 ± 2.8 Exp^e^: 60.9 ± 3.4 PassCon: 58.9 ± 3.7	Exp^d^: 1.78 ± 0.21 Exp^e^: 1.77 ± 0.31 PassCon: 1.78 ± 0.32	2 weeks	High	B&U	2	10	30–40 cm	48–144	No	More reps	January to April
Jlid et al. [47]	Exp: 36.5 ± 5.1 AtcCon: 34.2 ± 3.6	Exp: 1.43 ± 0.10 AtcCon:1.42 ± 0.04	ND	Normal	B&U	2	8	20–30 cm	54–124	No	Sets and reps	March-April
Jlid et al. [59]	Exp: 67.6 ± 5.9 AtcCon: 69.2 ± 5.8	Exp: 1.76 ± 0.05 AtcCon: 1.76 ± 0.06	ND	Moderate	B&U	2	6	30–50 cm	140–216	No	Sets and reps	March-April
Porrati-Paladino et al. [49]	Exp: 61.83 ± 9.17 ActCon: 66.16 ± 15.81	Exp: 23.07 ± 3.41 ActCon: 25.07 ± 5.90	ND	Moderate	B&U	3	6	ND	180	Eccentric; RT; plyometric	Sets and reps	ND
Ramirez-Campillo et al. [57]	Exp^a^: 43.5 ± 14.9 Exp^b^: 45.0 ± 9.3 Exp^c^: 42.2 ± 16.9 PassCon: 41.8 ± 12.7	Exp^a^: 146 ± 13.7 Exp^b^: 147 ± 11.1 Exp^c^: 144 ± 17.5 PassCon: 143 ± 17.7	6 weeks	Normal	Exp^a^: B Exp^b^: U Exp^c^: B&U	2	6	20 cm	120–240	No	Reps	Middle-season
Ramirez-Campillo et al. [24]	Exp^c,h^:40.0 ± 5.9 Exp^c,i^: 44.6 ± 11.0 Exp^c,j^: 40.1 ± 12.8 PassCon: 42.2 ± 16.9	Exp^c,h^: 144 Exp^c,i^: 150 Exp^c,j^: 141 PassCon: 146	6 weeks	Moderate	B&U	3	8	NA	ND	No	No	October – November
Trecoci et al. [43]	All, 48.8 ± 0.40	All, 1.53 ± 0.05	4.56 ± 0.66	Moderate	B&U	3	8	NA	ND	No	No	October – November

BH: box height for plyometric drop jumps (cm); BM: body mass (kg); Comb: combined; F: female; Freq: frequency of training (days/week); H: height of participants (cm); M: male; PO: progressive overload, in the form of either volume (i.e., V), intensity (i.e., I), type of drill (i.e., T), or a combination of these; TJ: total plyometric jumps; TP: training period of the season; Wk: weeks of training.

* Fitness level: high, for professional/elite athletes with regular enrollment in national and/or international competitions, highly trained participants with > 10 training hours per week or > 6 training sessions per week and a regularly scheduled official and friendly competitions. Moderate, for non-elite/professional athletes, with a regular attendance in regional and/or national competitions, between 5 and 9.9 training hours per week or 3–5 training sessions per week and a regularly scheduled official and friendly competitions. Normal, for recreational athletes with < 5 training hours per week with sporadic competitions’ participation, and for physically active participants and school-age youths regularly involved in physical education classes;

a : bilateral plyometric;

b : unilateral plyometric training;

c : combined bilateral and unilateral plyometric training group;

d : unloaded;

e : with load;

f : combined agility and plyometric;

g : combined balance and plyometric;

h : vertical plyometric;

i :horizontal plyometric;

j : combined vertical and horizontal plyometric;

l: combined strength, balance, agility and plyometric; M: male; F: female; Exp: experimental group; ActCon: active control group; PassCon: passive control group without experimental approach.

### Methodological quality

Using the PEDro checklist it was possible to determine that 2 of the studies were classified with 6 points, 4 studies with 7 points and 2 studies with eight points (Table 4).

**TABLE 4:** Methodological assessment with Physiotherapy Evidence Database (PEDro) scale

Reference	1	2	3	4	5	6	7	8	9	10	Score
Drouzas et al. [58]	Yes	Yes	Yes	No	No	No	Yes	No	Yes	Yes	Six
Hammami et al. [50]	Yes	Yes	Yes	No	Yes	No	Yes	No	Yes	Yes	Seven
Jlid et al. [47]	Yes	Yes	Yes	No	No	Yes	Yes	Yes	Yes	Yes	Eight
Jlid et al. [59]	Yes	Yes	Yes	No	No	Yes	Yes	Yes	Yes	Yes	Eight
Porrati-Paladino et al. [49]	Yes	Yes	Yes	No	No	Yes	Yes	No	Yes	Yes	Seven
Ramirez-Campillo et al. [57]	Yes	Yes	Yes	No	No	No	Yes	Yes	Yes	Yes	Seven
Ramirez-Campillo et al. [24]	Yes	Yes	Yes	No	No	No	Yes	Yes	Yes	Yes	Seven
Trecoci et al. [43]	Yes	Yes	No	No	No	No	Yes	No	Yes	Yes	Six

*: PEDRro scale items number; **: the total number of points from a possible maximal of 10; N.º1: eligibility criteria were specified; N.º2: subjects were randomly allocated to groups; N.º3: allocation was concealed; N.º4: the groups were similar at baseline regarding the most important prognostic indicators; N.º5: there was blinding of all subjects; N.º6: there was blinding of all therapists who administered the therapy; N.º7: there was blinding of all assessors who measured at least one key outcome; N.º8: measures of at least one key outcome were obtained from more than 85% of the subjects initially allocated to groups; N.º9: all subjects for whom outcome measures were available received the treatment or control condition as allocated or, where this was not the case, data for at least one key outcome was analyzed by “intention to treat”; N.º10: the results of between-group statistical comparisons are reported for at least one key outcome; and N.º11: the study provides both point measures and measures of variability for at least one key outcome.

### PJT vs. control: effects on dynamic balance

A summary of the included studies and results of anterior, posteromedial and postero-lateral dynamic balance before and after PJT-based intervention and control groups are provided in Tables 5, 6 and 7, respectively. Table 8 presents summary of the composites of dynamic balance.

**TABLE 5:** Summary of the included studies and results of dynamic balance (anterior) before and after intervention.

Study	Group	Laterality	Sex	TS	N	Before (left leg) Mean ± SD	After (left leg) Mean ± SD	After-before (left leg) (Δ%)	Before (right leg) Mean ± SD	After (right leg) Mean ± SD	After-before (right leg) (Δ%)
Hammami et al. [50]	Exp^d^	B&U	M	20	12	82 ± 8	85 ± 5	3.7	82 ± 8	86 ± 7	5.8
Hammami et al. [50]	Exp^e^	B&U	M	20	14	88 ± 10	93 ± 10	5.7	86 ± 8	91 ± 8	4.9
Hammami et al. [50]	ActCon	-	M	20	12	84 ± 7	87 ± 7	3.6	83 ± 8	83 ± 6	0.0
Jlid et al. [47]	Exp	B&U	M	16	14	65.5 ± 4.2	70.2 ± 3.6	7.3	80.9 ± 3.6	83.9 ± 3.7	3.8
Jlid et al. [47]	ActCon	-	M	16	14	67.7 ± 4.1	67.0 ± 4.4	-1.0	83.1 ± 1.7	83.3 ± 1.9	0.3
Jlid et al. [59]	Exp	B&U	M	12	14	74.4 ± 5.1	75.8 ± 4.7	1.8	74.0 ± 3.8	75.7 ± 3.4	2.3
Jlid et al. [59]	ActCon	-	M	12	13	72.6 ± 3.4	72.6 ± 3.8	0.02	71.2 ± 3.9	71.8 ± 3.8	0.8
Porrati-Paladino et al. [49]	Exp	B&U	F	18	9	59.7 ± 4.4	62.8 ± 4.1	5.2	60.2 ± 3.7	63.1 ± 4.7	4.8
Porrati-Paladino et al. [49]	ActCon	-	F	18	8	55.0 ± 4.0	58.4 ± 4.7	6.2	55.3 ± 3.8	61.3 ± 5.2	10.8

B: bilateral; U: unilateral; B&U: bilateral and unilateral; ActCon: active control; PassCon: passive control; TS: total sessions;

d : unloaded;

e : with load;

f: combined agility and plyometricM: male; F: female; Exp: experimental group; ActCon: active control group; PassCon: passive control group without experimental approach.

**TABLE 6:** Summary of the included studies and results of dynamic balance (posterior-medial) before and after intervention.

Study	Group	Laterality	Sex	TS	N	Before (left leg) Mean ± SD	After (left leg) Mean ± SD	After-before (left leg) (Δ%)	Before (right leg) Mean ± SD	After (right leg) Mean ± SD	After-before (right leg) (Δ%)
Hammami et al. [50]	Exp^d^	B&U	M	20	12	109 ± 12	108 ± 10	-0.9	106 ± 10	110 ± 9	3.8
Hammami et al. [50]	Exp^e^	B&U	M	20	14	114 ± 6	121 ± 5	6.1	111 ± 7	118 ± 5	6.3
Hammami et al. [50]	ActCon	-	M	20	12	113 ± 6	112 ± 7	-0.9	109 ± 8	112 ± 7	2.8
Jlid et al. [47]	Exp	B&U	M	16	14	80.8 ± 3.1	84.0 ± 2.3	3.9	74.9 ± 4.2	79.6 ± 3.9	6.39
Jlid et al. [47]	ActCon	-	M	16	14	82.5 ± 2.3	81.6 ± 2.6	-1.0	77.6 ± 4.1	77.7 ± 4.4	0.07
Jlid et al. [59]	Exp	B&U	M	12	14	80.2 ± 5.4	81.0 ± 5.6	1.2	74.1 ± 5.5	75.1 ± 5.1	1.41
Jlid et al. [59]	ActCon	-	M	12	13	78.5 ± 4.3	78.5 ± 4.4	-0.04	75.5 ± 7.0	76.2 ± 7.0	0.31
Porrati-Paladino et al. [49]	Exp	B&U	F	18	9	59.2 ± 6.2	64.6 ± 7.1	9.1	64.2 ± 7.2	67.56 ± 5.5	5.2
Porrati-Paladino et al. [49]	ActCon	-	F	18	8	55.6 ± 6.9	64.3 ± 6.5	15.6	57.4 ± 10.1	63.88 ± 7.7	11.3

B: bilateral; U: unilateral; B&U: bilateral and unilateral; ActCon: active control; PassCon: passive control; TS: total sessions;

d : unloaded;

e : with load;

f: combined agility and plyometric; M: male; F: female; Exp: experimental group; ActCon: active control group; PassCon: passive control group without experimental approach.

**TABLE 7:** Summary of the included studies and results of dynamic balance (posterior-lateral) before and after intervention.

Study	Group	Latera-lity	Sex	TS	N	Before (left leg) Mean ± SD	After (left leg) Mean ± SD	After-before (left leg) (Δ%)	Before (right leg) Mean ± SD	After (right leg) Mean ± SD	After-before (right leg) (Δ%)
Hammami et al. [50]	Exp^d^	B&U	M	20	12	55 ± 11	53 ± 11	-3.6	53 ± 12	53 ± 12	0.0
Hammami et al. [50]	Exp^e^	B&U	M	20	14	53 ± 8	55 ± 8	3.8	55 ± 9	60 ± 9	9.1
Hammami et al. [50]	ActCon	-	M	20	12	51 ± 11	47 ± 11	-7.8	52 ± 10	52 ± 10	0.0
Jlid et al. [47]	Exp	B&U	M	16	14	89.5 ± 6.5	89.0 ± 7.6	-0.1	82.3 ± 7.1	86.2 ± 5.4	4.93
Jlid et al. [47]	ActCon	-	M	16	14	91.0 ± 2.9	89.0 ± 4.9	-2.2	84.4 ± 8.8	83.9 ± 8.6	−0.59
Jlid et al. [59]	Exp	B&U	M	12	14	87.0 ± 4.7	88.8 ± 4.2	2.0	86.1 ± 4.0	87.8 ± 3.7	1.96
Jlid et al. [59]	ActCon	-	M	12	13	87.9 ± 3.2	88.5 ± 3.4	0.7	86.7 ± 3.3	87.3 ± 3.5	1.83
Porrati-Paladino et al. [49]	Exp	B&U	F	18	9	70.4 ± 9.2	71.11 ± 8.2	1.0	64.1 ± 10.8	68.6 ± 10.9	7.0
Porrati-Paladino et al. [49]	ActCon	-	F	18	8	65.4 ± 7.2	69.9 ± 6.7	6.9	63.1 ± 8.2	71.13 ± 5.4	12.7

B: bilateral; U: unilateral; B&U: bilateral and unilateral; ActCon: active control; PassCon: passive control; TS: total sessions;

d : unloaded;

e : with load;

f: combined agility and plyometric; M: male; F: female; Exp: experimental group; ActCon: active control group; PassCon: passive control group without experimental approach.

**TABLE 8:** Summary of the included studies and results of dynamic balance (composite score) before and after intervention.

Study	Group	Latera-lity	Sex	TS	N	Before (left leg) Mean ± SD	After (left leg) Mean ± SD	After-before (left leg) (Δ%)	Before (right leg) Mean ± SD	After (right leg) Mean ± SD	After-before (right leg) (Δ%)
Trecroci et al. [43]	Exp	B&U	M	24	12	139.1 ± 18.6	143.0 ± 17.8	2.8	140.5 ± 16.3	144.4 ± 17.4	2.8
Trecroci et al. [43]	ActCon	-	M	24	12	133.8 ± 11.9	134.7 ± 12.2	0.7	133.1 ± 13.0	134.2 ± 11.7	0.8

**FIG. 2:**
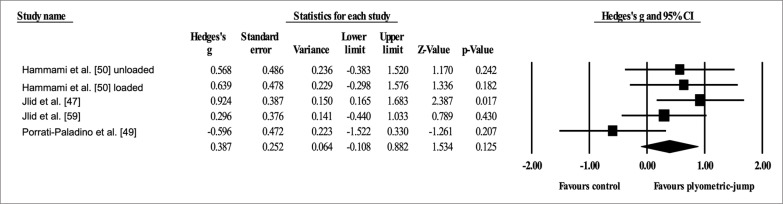
Forest plot of changes in dynamic anterior balance with the right leg after participating in plyometric-jump training programmes compared to active control conditions. Values shown are effect sizes (Hedges’s g) with 95% confidence intervals (CI). The size of the plotted squares reflects the statistical weight of each study. The black diamond reflects the overall result.

B: bilateral; U: unilateral; B&U: bilateral and unilateral; ActCon: active control; PassCon: passive control; TS: total sessions; M: male; Exp: experimental group; ActCon: active control group;

Four studies provided data for dynamic anterior balance with the right leg, involving five experimental and four active control groups (pooled *n* = 110). Results showed a small effect of PJT on dynamic anterior balance with the right leg (ES = 0.39; 95% CI = -0.11 to 0.88; *p =* 0.125; *I* ^2^ = 40.5%; Egger’s test *p* = 0.602; Figure 2) when compared to active controls.

**FIG. 3:**
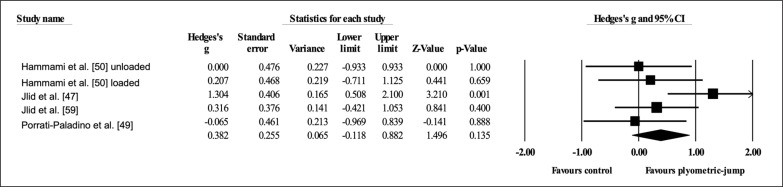
Forest plot of changes in dynamic anterior balance with the left leg after participating in plyometric-jump training programmes compared to active control conditions. Values shown are effect sizes (Hedges’s g) with 95% confidence intervals (CI). The size of the plotted squares reflects the statistical weight of each study. The black diamond reflects the overall result.

Four studies provided data for dynamic anterior balance with the left leg, involving five experimental and four active control groups (pooled *n* = 110). Results showed a small effect of PJT on dynamic anterior balance with the left leg (ES = 0.38; 95% CI = -0.12 to 0.88; *p =* 0.135; *I* ^2^ = 42.0%; Egger’s test *p* = 0.383; Figure 3) when compared to active controls.

**FIG. 4:**
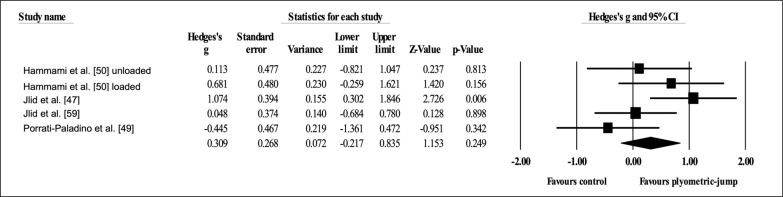
Forest plot of changes in dynamic postero-medial balance with the right leg after participating in plyometric-jump training programmes compared to active control conditions. Values shown are effect sizes (Hedges’s g) with 95% confidence intervals (CI). The size of the plotted squares reflects the statistical weight of each study. The black diamond reflects the overall result.

Four studies provided data for dynamic postero-medial balance with the right leg, involving five experimental and four active control groups (pooled *n* = 110). Results showed a small effect of PJT on dynamic postero-medial balance with the right leg (ES = 0.31; 95% CI = -0.22 to 0.84; *p =* 0.249; *I* ^2^ = 47.5%; Egger’s test *p* = 0.696; Figure 4) when compared to active controls.

**FIG. 5:**
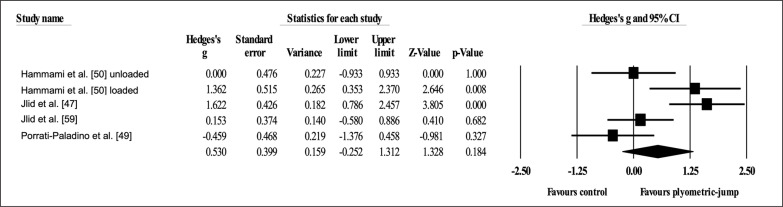
Forest plot of changes in dynamic postero-medial balance with the left leg after participating in plyometric-jump training programmes compared to active control conditions. Values shown are effect sizes (Hedges’s g) with 95% confidence intervals (CI). The size of the plotted squares reflects the statistical weight of each study. The black diamond reflects the overall result.

Four studies provided data for dynamic postero-medial balance with the left leg, involving five experimental and four active control groups (pooled *n* = 110). Results showed a small effect of PJT on dynamic postero-medial balance with the left leg (ES = 0.53; 95% CI = -0.25 to 1.31; *p =* 0.184; *I* ^2^ = 74.8%; Egger’s test *p* = 0.849; Figure 5) when compared to active controls.

**FIG. 6:**
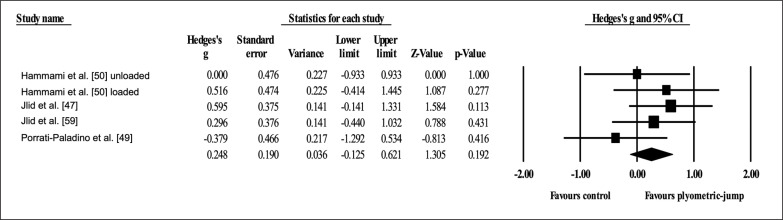
Forest plot of changes in dynamic postero-lateral balance with the right leg after participating in plyometric-jump training programmes compared to active control conditions. Values shown are effect sizes (Hedges’s g) with 95% confidence intervals (CI). The size of the plotted squares reflects the statistical weight of each study. The black diamond reflects the overall result.

Four studies provided data for dynamic postero-lateral balance with the right leg, involving five experimental and four active control groups (pooled *n* = 110). Results showed a small effect of PJT on dynamic postero-lateral balance with the right leg (ES = 0.25; 95% CI = -0.13 to 0.62; *p =* 0.192; *I* ^2^ = 0.0%; Egger’s test *p* = 0.335; Figure 6) when compared to active controls.

**FIG. 7:**
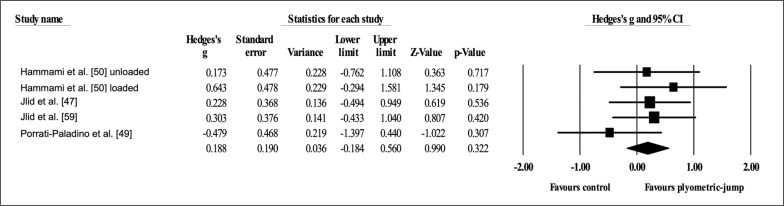
Forest plot of changes in dynamic postero-lateral balance with the left leg after participating in plyometric-jump training programmes compared to active control conditions. Values shown are effect sizes (Hedges’s g) with 95% confidence intervals (CI). The size of the plotted squares reflects the statistical weight of each study. The black diamond reflects the overall result.

Four studies provided data for dynamic postero-lateral balance with the left leg, involving five experimental and four active control groups (pooled *n* = 110). Results showed a trivial effect of PJT on dynamic postero-lateral balance with the left leg (ES = 0.19; 95% CI = -0.18 to 0.56; *p =* 0.322; *I* ^2^ = 0.0%; Egger’s test *p* = 0.769; Figure 7) when compared to active controls.

### PJT vs. control: effects on static balance

A summary of the included studies and results of static balance reported before and after PJT-based intervention and control groups are provided in Table 9.

**TABLE 9:** Summary of the included studies and results of static balance before and after intervention.

Study	Group	Laterality	Sex	TS	N	Before Mean ± SD	After Mean ± SD	After-before (Δ%)
Drouzas et al. [58]	Exp^a^	U	M	20	23	20 ± 8	17 ± 5	-15.0
Drouzas et al. [58]	Exp^b^	B	M	20	23	20 ± 8	17 ± 6	-15.0
Drouzas et al. [58]	ActCon	-	M	-	22	20 ± 7	17 ± 5	-15.0
Hammami et al. [50]	Exp^d^	B&U	M	20	12	2.24 ± 0.76	7.78 ± 4.50	247.3
Hammami et al. [50]	Exp^e^	B&U	M	20	14	3.37 ± 2.94	4.86 ± 3.42	44.2
Hammami et al. [50]	ActCon	-	M	20	12	2.16 ± 0.61	3.35 ± 2.53	55.1
Ramirez-Campillo et al. [57]	Exp^a^	B	M	12	12	0.50 ± 0.18	0.46 ± 0.08	-8.1
Ramirez-Campillo et al. [57]	Exp^b^	U	M	12	16	0.45 ± 0.20	0.40 ± 0.17	-9.8
Ramirez-Campillo et al. [57]	Exp^c^	B&U	M	12	12	0.51 ± 0.17	0.40 ± 0.10	-19.6
Ramirez-Campillo et al. [57]	PassCon	-	M	24	14	0.50 ± 0.15	0.50 ± 0.15	-1.5
Ramirez-Campillo et al. [24]	Exp^c,h^	B&U	M	24	10	0.42 ± 0.11	0.38 ± 0.10	-9.9
Ramirez-Campillo et al. [24]	Exp^c,i^	B&U	M	24	10	0.52 ± 0.20	0.45 ± 0.16	-11.2
Ramirez-Campillo et al. [24]	Exp^c,j^	B&U	M	24	10	0.47 ± 0.17	0.38 ± 0.10	-16.2
Ramirez-Campillo et al. [24]	PassCon	-	M	24	10	0.50 ± 0.13	0.47 ± 0.10	-4.3

B: bilateral; U: unilateral; B&U: bilateral and unilateral; ActCon: active control; PassCon: passive control; TS: total sessions;

a : bilateral plyometric;

b : unilateral plyometric training;

c : combined bilateral and unilateral plyometric training group;

d : unloaded;

e : with load;

f : combined agility and plyometric;

g : combined balance and plyometric;

h : vertical plyometric;

i :horizontal plyometric;

j : combined vertical and horizontal plyometric;

l: combined strength, balance, agility and plyometric; M: male; F: female; Exp: experimental group; ActCon: active control group; PassCon: passive control group without experimental approach.

**FIG. 8:**
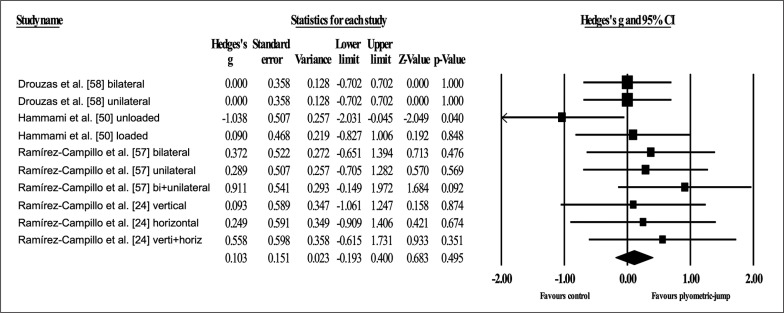
Forest plot of changes in static balance after participating in plyometric-jump training programmes compared to active control conditions. Values shown are effect sizes (Hedges’s g) with 95% confidence intervals (CI). The size of the plotted squares reflects the statistical weight of each study. The black diamond reflects the overall result.

Four studies provided data for static balance, involving ten experimental and four active control groups (pooled *n* = 200). Results showed a trivial effect of PJT on static balance (ES = 0.10; 95% CI = -0.19 to 0.40; *p =* 0.495; *I*^2^ = 0.0%; Egger’s test *p* = 0.424; Figure 8) when compared to active controls.

## DISCUSSION

The purpose of this systematic review with meta-analysis was to assess the effects of PJT programs on dynamic and static balance in soccer players. The main finding of this review is that PJT has no significant advantage over active control conditions in terms of improving soccer players’ dynamic or static balance, although small magnitudes of change in favor of PJT were found.

### Effects of PJT on dynamic balance

Dynamic balance is considered a key factor in soccer performance since it helps players to maintain a stable center of gravity when executing sport-specific movements [42]. For example, a player must exhibit excellent balance to keep their body stable while performing combinations of upper- and lower-body movements during the flight, landing, and take-off phases of jumps, which are crucial for successful participation in soccer [43].

In this regard, PJT seems to be an essential training method for improving soccer players’ dynamic balance capacity by improving their neuromuscular control [44], proactive and/or feed-forward adjustments [45], and the sensitivity of afferent feedback pathway during exercise [46].

Despite this, the results obtained in our systematic review with meta-analysis revealed no significant improvements in dynamic balance after applying PJT in comparison to control groups in all movement planes, although some small changes in favor of PJT were detected. However, it could be that the age and sex of participants, as well as the duration of interventions, influenced the results.

Specifically, significant improvements in all movement planes were observed in the youngest players (i.e., 11.8 ± 0.4 years) [47], possibly because adaptations are achieved more rapidly at early ages [48]. Nevertheless, Porrati-Paladino and Cuesta-Barriuso [49] did not observe significant improvements in any movement plane after the application of a multi-direction PJT in U23 female players over six weeks. Finally, Hammami et al. [50] revealed better results in an ankle-loaded group, highlighting the necessity of stressing the implicated muscles beyond their normal capacity to obtain significant adaptative responses [51].

Concerning the observed results, PJT seems to be a key strategy for preventing decreases in the dynamic balance capacity of soccer players, which is associated with an increase in injury risk [47]. However, several factors such as sex, age, and intervention duration must be considered when PJT is prescribed as a balance-improvement method.

### Effects of PJT on static balance

In addition to dynamic balance, players must achieve and maintain adequate levels of static balance to resist falling and act efficiently during soccer practice [52]. Specifically, players must maintain their balance and posture before executing regular soccer actions such as sprinting or jumping in order to improve their efficiency or aestheticism in these movements [53].

In this sense, the PJT facilitates several neuromuscular adaptations in the lower limbs responsible for static balance [54]. Such adaptations include improvements in inter-muscular coordination, muscle size and architecture, changes in single-fiber mechanics, and changes in muscle-tendon mechanical-stiffness [55], which could improve physical performance and minimize injury risk [56].

Our study showed that PJT has no significant effect on static balance in comparison to other active control conditions, possibly due to the differences in the purposes of the PJTs used across studies (e.g., bilateral, unilateral, or combined; vertical, horizontal, or combined) and in the participants’ age (which ranged from eight to 16 years).

Nevertheless, the individualized analysis of the selected studies revealed some important findings. On the one hand, most of participants improved their static balance capacity, albeit without presenting significant differences when compared to control groups. As such, PJT could be an interesting method for maintaining this capacity while also improving other physical qualities (i.e., jumping and sprinting) [26].

On the other hand, Ramirez-Campillo et al. [24;57] observed that, compared to the control group, significant improvements in static balance occurred when combined PJT programs were applied in terms of laterality (i.e., bilateral + unilateral exercises) and movement plane (i.e., vertical + horizontal exercises). Additionally, Ramírez-Campillo et al. [24] stated the importance of the specificity of the exercise selection, as these authors observed better results for the anterior-posterior plane, which could be associated with the anterior-posterior training nature purposed for each group. Finally, coinciding with aforementioned results on dynamic balance, Hammami et al. [50] reported greater improvements in static balance for an ankle-loaded group. The results obtained in this investigation suggest that using a combined PJT improves the static balance of soccer players. This finding highlights the necessity of applying specific exercises when the goal is to improve balance on a specific plane.

### Study limitations, future research, and practical applications

This systematic review with meta-analysis has several limitations. Most notably, the heterogeneity sample included in the selected studies is characterized by a broad age range (8–23 years), different sexes, and varying skill levels of participants (amateur, sub-elite, and elite). Another limitation is the small number of researches related to the effects of PJT on dynamic balance (i.e., only three investigations with five different cohorts) and static balance (i.e., only three investigations with 10 different cohorts using many PJT programs). Therefore, strength and conditioning specialists should consider the conclusions and practical applications with caution.

Due to the promising benefits of PJT on the maintenance/improvement of balance in soccer players, future studies should analyze the same PJT (per the recommendations presented in this study) in different populations in order to be able to generalize the results. Moreover, considerations about the day of PJT implementation and type of exercises should be considered [60]. Additionally, longer PJT programs are required to validate our findings regarding the effects of these programs on balance. In fact, it is expectable that PJT stimulate proprioceptors (e.g., muscle spindle, Goldi tendon organ and mechanoreceptors located in join capsules and ligaments) and improve the neural efficiency through enhancement of neuromuscular coordination by possibly also improving balance [61].

Concerning the observed findings from this systematic review with meta-analysis, some practical indications must be considered when PJT programs were prescribed with a focus on players’ balance:

– Combined PJT in terms of laterality (i.e., bilateral + unilateral exercises) and movement plane (i.e., vertical + horizontal exercises) must be applied.
– Specificity in selected exercises is required to achieve selected improvements in terms of movement planes.
– Ankle overload could improve dynamic and static balance in soccer players.
– PJT on unstable surfaces must be included to improve the cocontraction of the lower extremity muscles and changes in proprioception and neuromuscular control.

## CONCLUSIONS

The present systematic review suggests that PJT provides no significant benefits in terms of dynamic and static balance when compared to other active control programmes (specific soccer training). More studies should be conducted on this topic, specifically considering the effect of several moderators, such as the laterality of the PJT.

## Funding

Filipe Manuel Clemente: This work is funded by Fundação para a Ciência e Tecnologia/ Ministério da Ciência, Tecnologia e Ensino Superior through national funds and when applicable co-funded EU funds under the project UIDB/50008/2020. No other specific sources of funding were used to assist in the preparation of this article.

## Conflicts of interest/Competing interests

The authors declare that they have no conflicts of interest relevant to the content of this review.

## References

[1] Stolen T, Chamari K, Castagna C, Wisloff U. Physiology of soccer: an update. Sport Med. 2005; 35(6):501–36. 10.2165/00007256-200535060-00004 15974635

[2] Dolci F, Hart NH, Kilding AE, Chivers P, Piggott B, Spiteri T. Physical and Energetic Demand of Soccer. Strength Cond J. 2020; 42(3):70–7.

[3] Aquino R, Puggina EF, Alves IS, Garganta J. Skill-related performance in soccer: a systematic review. Hum Mov. 2017; 18(5).

[4] Clemente FM, Sarmento H. The effects of small-sided soccer games on technical actions and skills: A systematic review. Hum Mov. 2020; 21(3):100–19.

[5] Wing CE, Turner AN, Bishop CJ. Importance of Strength and Power on Key Performance Indicators in Elite Youth Soccer. J Strength Cond Res. 2020; 34(7):2006–14. 29373431 10.1519/JSC.0000000000002446

[6] Buchheit M, Mendez-Villanueva A, Simpson BM, Bourdon PC. Match Running Performance and Fitness in Youth Soccer. Int J Sports Med. 2010; 31(11):818–25. 20703978 10.1055/s-0030-1262838

[7] Chew-Bullock TS-Y, Anderson DI, Hamel KA, Gorelick ML, Wallace SA, Sidaway B. Kicking performance in relation to balance ability over the support leg. Hum Mov Sci. 2012; 31(6):1615–23. 22939850 10.1016/j.humov.2012.07.001

[8] Śliwowski R, Grygorowicz M, Wieczorek A, Jadczak Ł. The relationship between jumping performance, isokinetic strength and dynamic postural control in elite youth soccer players. J Sport Med Phys Fit. 2018; 58(9):1226–33. 10.23736/S0022-4707.17.07289-9 28639440

[9] Gualtieri D, Cattaneo A, Sarcinella R, Cimadoro G, Alberti G. Relationship between balance capacity and jump ability in amateur soccer players of different ages. Sport Sci Health. 2008; 3(3):73–6.

[10] Hrysomallis C. Relationship Between Balance Ability, Training and Sports Injury Risk. Sport Med. 2007; 37(6):547–56. 10.2165/00007256-200737060-00007 17503879

[11] Gonell AC, Romero JAP, Soler LM. Relationship between the Y balance test scores and soft tissue injury incidence in a soccer team. Int J Sports Phys Ther. 2015; 10(7):955–66. 26673848 PMC4675196

[12] Winter DA, Patla AE, Frank JS. Assessment of balance control in humans. Med prog technol. 1990; 16(1–2):31–51. 2138696

[13] Bressel E, Yonker JC, Kras J, Heath EM. Comparison of Static and Dynamic Balance in Female Collegiate Soccer, Basketball, and Gymnastics Athletes. J Athl Train. 2007; 42(1):42–46. 17597942 PMC1896078

[14] Peterka RJ. Sensory integration for human balance control. In: Handbook of Clinical Neurology. Elsevier; 2018. p. 27–42. 10.1016/B978-0-444-63916-5.00002-1 30482320

[15] Hoch MC, Staton GS, McKeon PO. Dorsiflexion range of motion significantly influences dynamic balance. J Sci Med Sport. 2011; 14(1):90–2. 20843744 10.1016/j.jsams.2010.08.001

[16] Zech A, Hübscher M, Vogt L, Banzer W, Hänsel F, Pfeifer K. Balance Training for Neuromuscular Control and Performance Enhancement: A Systematic Review. J Athl Train. 2010; 45(4):392–403. 20617915 10.4085/1062-6050-45.4.392 PMC2902034

[17] Pau M, Arippa F, Leban B, Corona F, Ibba G, Todde F, et al. Relationship between static and dynamic balance abilities in Italian professional and youth league soccer players. Phys Ther Sport. 2015; 16(3):236–41. 25869425 10.1016/j.ptsp.2014.12.003

[18] Pau M, Ibba G, Leban B, Scorcu M. Characterization of Static Balance Abilities in Elite Soccer Players by Playing Position and Age. Res Sport Med [Internet]. 2014; 22(4):355–67. 10.1080/15438627.2014.944302 25295474

[19] Munro AG, Herrington LC. Between-session reliability of the star excursion balance test. Phys Ther Sport. 2010; 11(4):128–32. 21055706 10.1016/j.ptsp.2010.07.002

[20] Coughlan GF, Fullam K, Delahunt E, Gissane C, Caulfield BM, Sci M. A Comparison Between Performance on Selected Directions of the Star Excursion Balance Test and the Y Balance Test. J Athl Train. 2012; 47(4):366–71. 22889651 10.4085/1062-6050-47.4.03 PMC3396295

[21] Davlin CD. Dynamic Balance in High Level Athletes. Percept Mot Skills. 2004; 98(3_suppl):1171–6. 15291203 10.2466/pms.98.3c.1171-1176

[22] Platzer H-P, Raschner C, Patterson C. Performance-determining physiological factors in the luge start. J Sports Sci. 2009; 27(3):221–6. 19156559 10.1080/02640410802400799

[23] Kümmel J, Kramer A, Giboin L-S, Gruber M. Specificity of Balance Training in Healthy Individuals: A Systematic Review and Meta-Analysis. Sport Med. 2016; 46(9):1261–71. 10.1007/s40279-016-0515-z 26993132

[24] Ramírez-Campillo R, Gallardo F, Henriquez-Olguín C, Meylan CMP, Martínez C, Álvarez C, et al. Effect of Vertical, Horizontal, and Combined Plyometric Training on Explosive, Balance, and Endurance Performance of Young Soccer Players. J Strength Cond Res. 2015; 29(7):1784–95. 25559903 10.1519/JSC.0000000000000827

[25] Ramirez-Campillo R, Sanchez-Sanchez J, Romero-Moraleda B, Yanci J, García-Hermoso A, Manuel Clemente F. Effects of plyometric jump training in female soccer player’s vertical jump height: A systematic review with meta-analysis. J Sports Sci. 2020; 38(13):1475–87. 32255389 10.1080/02640414.2020.1745503

[26] Ramirez-Campillo R, Castillo D, Raya-González J, Moran J, de Villarreal ES, Lloyd RS. Effects of Plyometric Jump Training on Jump and Sprint Performance in Young Male Soccer Players: A Systematic Review and Meta-analysis. Sport Med. 2020; 50(12):2125–43. 10.1007/s40279-020-01337-1 32915430

[27] Wang Y-C, Zhang N. Effects of plyometric training on soccer players. Exp Ther Med. 2016; 12(2):550–4. 27446242 10.3892/etm.2016.3419 PMC4950532

[28] Taube W, Leukel C, Gollhofer A. How Neurons Make Us Jump. Exerc Sport Sci Rev. 2012; 40(2):106–15. 22089697 10.1097/JES.0b013e31824138da

[29] Ramirez-Campillo R, Álvarez C, García-Hermoso A, Ramírez-Vélez R, Gentil P, Asadi A, et al. Methodological Characteristics and Future Directions for Plyometric Jump Training Research: A Scoping Review. Sport Med [Internet]. 2018; 48(5):1059–81. 10.1007/s40279-018-0870-z 29470823

[30] Mikołajec K, Maszczyk A, Chalimoniuk M, Langfort J, Gołaś A, Zajc A. The influence of strength exercises of the lower limbs on postural stability: A possible role of the autonomic nervous system. Isokinet Exerc Sci. 2017; 25(2):79–89.

[31] Green S, Higgins J. Cochrane handbook for systematic reviews of interventions. NJ, USA: John Wiley & Sons: Hoboken; 2005.

[32] Moher D, Liberati A, Tetzlaff J, Altman DG. Preferred Reporting Items for Systematic Reviews and Meta-Analyses: The PRISMA Statement. PLoS Med. 2009; 6(7):e1000097. 19621072 10.1371/journal.pmed.1000097 PMC2707599

[33] Rico-González M, Pino-Ortega J, Clemente FM, Arcos AL. Guidelines for performing systematic reviews in sports science. 2022;39(2):463–471. 10.5114/biolsport.2022.106386 PMC8919872 35309539

[34] Collaboration C. Data Extraction Template for Included Studies. 2016.

[35] Deeks JJ, Higgins JP, Altman DG. Analysing data and undertaking meta-analyses. In: Higgins JP, Green S, editors. Cochrane Handbook for Systematic Reviews of Interventions. The Cochrane Collaboration; 2008. p. 243–96.

[36] Kontopantelis E, Springate DA, Reeves D. A Re-Analysis of the Cochrane Library Data: The Dangers of Unobserved Heterogeneity in Meta-Analyses. Friede T, editor. PLoS One. 2013; 8(7):e69930. 23922860 10.1371/journal.pone.0069930 PMC3724681

[37] Hopkins WG, Marshall SW, Batterham AM, Hanin J. Progressive Statistics for Studies in Sports Medicine and Exercise Science. Med Sci Sport Exerc [Internet]. 2009; 41(1):3–13. 10.1249/MSS.0b013e31818cb278 19092709

[38] Higgins JPT, Thompson SG. Quantifying heterogeneity in a meta-analysis. Stat Med. 2002; 21(11):1539–58. 12111919 10.1002/sim.1186

[39] Egger M, Smith GD, Schneider M, Minder C. Bias in meta-analysis detected by a simple, graphical test. BMJ. 1997 Sep 13; 315(7109):629–34. 9310563 10.1136/bmj.315.7109.629 PMC2127453

[40] Duval S, Tweedie R. Trim and Fill: A Simple Funnel-Plot-Based Method of Testing and Adjusting for Publication Bias in Meta-Analysis. Biometrics. 2000; 56(2):455–63. 10877304 10.1111/j.0006-341x.2000.00455.x

[41] Shi L, Lin L. The trim-and-fill method for publication bias. Medicine. 2019; 98(23):e15987. 31169736 10.1097/MD.0000000000015987 PMC6571372

[42] Lockie RG, Schultz AB, Callaghan SJ, Jeffriess MD. The Relationship between Dynamic Stability and Multidirectional Speed. J Strength Cond Res. 2016 Nov; 30(11):3033–43. 23942170 10.1519/JSC.0b013e3182a744b6

[43] Trecroci A, Cavaggioni L, Caccia R, Alberti G. Jump rope training: Balance and motor coordination in preadolescent soccer players. J Sports Sci Med. 2015; 14(4):792–8. 26664276 PMC4657422

[44] Asadi A, Saez De Villarreal E, Arazi H. The Effects of Plyometric Type Neuromuscular Training on Postural Control Performance of Male Team Basketball Players. J Strength Cond Res. 2015 Jul; 29(7):1870–5. 25563677 10.1519/JSC.0000000000000832

[45] Paillard T. Sport-specific balance develops specific postural skills. Vol. 44, Sports Medicine. Springer International Publishing; 2014. p. 1019–20. 10.1007/s40279-014-0174-x PMC4072915 24668292

[46] Borghuis J, Hof AL, Lemmink KAPM. The importance of sensory-motor control in providing core stability: Implications for measurement and training. Vol. 38, Sports Medicine. Sports Med; 2008. p. 893–916. 18937521 10.2165/00007256-200838110-00002

[47] Jlid MC, Racil G, Coquart J, Paillard T, Bisciotti GN, Chamari K. Multidirectional Plyometric Training: Very Efficient Way to Improve Vertical Jump Performance, Change of Direction Performance and Dynamic Postural Control in Young Soccer Players. Front Physiol. 2019; 10. 31920686 10.3389/fphys.2019.01462 PMC6913538

[48] Haff GG, Nimphius S. Training Principles for Power. Strength Cond J. 2012; 34(6):2–12.

[49] Porrati-Paladino G, Cuesta-Barriuso R. Effectiveness of Plyometric and Eccentric Exercise for Jumping and Stability in Female Soccer Players—A Single-Blind, Randomized Controlled Pilot Study. Int J Environ Res Public Health. 2021; 18(1):294. 10.3390/ijerph18010294 PMC7796027 33401532

[50] Hammami M, Gaamouri N, Suzuki K, Aouadi R, Shephard RJ, Chelly MS. Effects of Unloaded vs. Ankle-Loaded Plyometric Training on the Physical Fitness of U-17 Male Soccer Players. Int J Environ Res Public Health. 2020; 17(21):7877. 10.3390/ijerph17217877 PMC7663380 33121121

[51] Issurin VB. Training transfer: Scientific background and insights for practical application. Vol. 43, Sports Medicine. Sports Med; 2013. p. 675–94. 23633165 10.1007/s40279-013-0049-6

[52] Paillard T, Noé F. Techniques and Methods for Testing the Postural Function in Healthy and Pathological Subjects. Vol. 2015, BioMed Research International. Hindawi Publishing Corporation; 2015. 10.1155/2015/891390 PMC4659957 26640800

[53] Paillard T. Relationship between sport expertise and postural skills. Vol. 10, Frontiers in Psychology. Frontiers Media S.A.; 2019. 10.3389/fpsyg.2019.01428 PMC6603331 31293483

[54] Paillard T. Plasticity of the postural function to sport and/or motor experience. Vol. 72, Neuroscience and Biobehavioral Reviews. Elsevier Ltd; 2017. p. 129–52. 10.1016/j.neubiorev.2016.11.015 27894829

[55] Markovic G, Mikulic P. Neuro-Musculoskeletal and Performance Adaptations to Lower-Extremity Plyometric Training. Sport Med. 2010; 40(10):859–95. 10.2165/11318370-000000000-00000 20836583

[56] Chimera NJ, Swanik KA, Swanik CB, Straub SJ. Effects of Plyometric Training on Muscle-Activation Strategies and Performance in Female Athletes. J Athl Train. 2004 Jan; 39(1):24–31. 15085208 PMC385258

[57] Ramirez-Campillo R, Burgos C, Henriques-Olguin C, Andrade D, Martinez C, Alvarez C, et al. Effect Of Unilateral, Bilateral, And Combined Plyometric Training On Explosive And Endurance Perfomance Of Young Soccer Players. J Strength Cond Res. 2015; 29(5):1317–28. 25474338 10.1519/JSC.0000000000000762

[58] Drouzas V, Katsikas C, Zafeiridis A, Jamurtas AZ, Bogdanis GC. Unilateral Plyometric Training is Superior to Volume-Matched Bilateral Training for Improving Strength, Speed and Power of Lower Limbs in Preadolescent Soccer Athletes. J Hum Kinet. 2020; 74(1):161–76. 33312284 10.2478/hukin-2020-0022 PMC7706637

[59] Jlid MC, Coquart J, Maffulli N, Paillard T, Bisciotti GN, Chamari K. Effects of in Season Multi-Directional Plyometric Training on Vertical Jump Performance, Change of Direction Speed and Dynamic Postural Control in U-21 Soccer Players. Front Physiol. 2020; 11:374. 32431621 10.3389/fphys.2020.00374 PMC7212831

[60] Weldon A, Duncan M, Turner A, Sampaio J, Noon M, Wong DP, Lai VW Contemporary practices of strength and conditioning coaches in professional soccer. Biol Sport. 2021; 38(3):377–390. 34475621 10.5114/biolsport.2021.99328 PMC8329977

[61] Davies G, Riemann BL, Manske R. Current concepts of plyometric exercise. Int J Sport Phy Ther. 2015; 10(6): 760–786. PMC4637913 26618058

